# Altering *Escherichia coli* envelope integrity by mimicking the lipoprotein RcsF

**DOI:** 10.1007/s00203-023-03733-3

**Published:** 2023-12-09

**Authors:** Moustafa A. TagElDein, Noha G. Mohamed, Yasser E. Shahein, Laila Ziko, Nahla A. Hussein

**Affiliations:** 1 Microbiology and Immunology Department, Faculty of Pharmacy, Cairo University, Cairo, Egypt; 2 Pharmaceutical Chemistry Department, Faculty of Pharmacy, Sphinx University, Assiut, Egypt; 3 Molecular Biology Department, Biotechnology Research Institute, National Research Centre, Cairo, Egypt; 4 Department of Biochemistry, School of Life and Medical Sciences, University of Hertfordshire Hosted By the Global Academic Foundation, R5 New Garden City, New Administrative Capital, Cairo, Egypt

**Keywords:** RcsF, Antibacterial, Antimicrobial peptide, Rcs system

## Abstract

*Escherichia coli* cell envelope is crucial for stress sensing and signal transduction, mediated by numerous protein–protein interactions to enable adaptation and survival. Interfering with these interactions might affect envelope integrity leading to bacterial death. The outer membrane lipoprotein (RcsF) is the stress sensor of the regulator of capsule synthesis (Rcs) phosphorelay that senses envelope threats. RcsF interacts with two essential proteins, IgaA (repressing the Rcs system) and BamA (inserting β-barrel proteins in the outer membrane). Disturbing RcsF interactions may alter Rcs signaling and/or membrane integrity thus affecting bacterial survival. Here, we derived the sequence of a peptide mimicking RcsF (RcsFmim), based on the in silico docking of RcsF with IgaA. Expression of *rcsF*mim caused 3-to-4-fold activation of the Rcs system and perturbation of the outer membrane. Both effects result in decreased *E. coli* growth rate. We anticipate that RcsFmim present a candidate for future antibacterial peptide development.

**Supplementary Information:**

The online version contains supplementary material available at 10.1007/s00203-023-03733-3.

## Introduction

The Gram-negative cell envelope is a unique and complex macromolecular structure. Formed of an inner membrane and an outer membrane separated by the periplasmic space where the peptidoglycan resides (Silhavy et al. 2010), it represents a formidable barrier to physical and chemical stressors. Cell envelope integrity is a keystone in bacterial survival, adaptation and homeostasis. Accordingly, cell envelope proteins which play key roles in response to environmental stressors and participate in key complexes and signaling pathways are promising targets for antimicrobial development. Disturbing or interfering with their function will likely lead to loss of membrane integrity and an overall negative effect on bacterial survival and adaptation.

RcsF, a 12 kDa outer membrane lipoprotein is a typical example of such proteins. It is the stress sensor of the Regulator of capsule synthesis (Rcs) system, a complex phosphorelay that sense and respond to cell envelope threats (Stout and Gottesman 1990; Majdalani and Gottesman 2005). The Rcs system functions in parallel to two other two component systems (TCSs), the Conjugative pilus expression (Cpx) and the Bacterial adaptive response (Bae) systems (Leblanc et al. 2011; Delhaye et al. 2016; Grabowicz and Silhavy 2017; Erin Wall et al.^2018^).

**Fig. 1:**
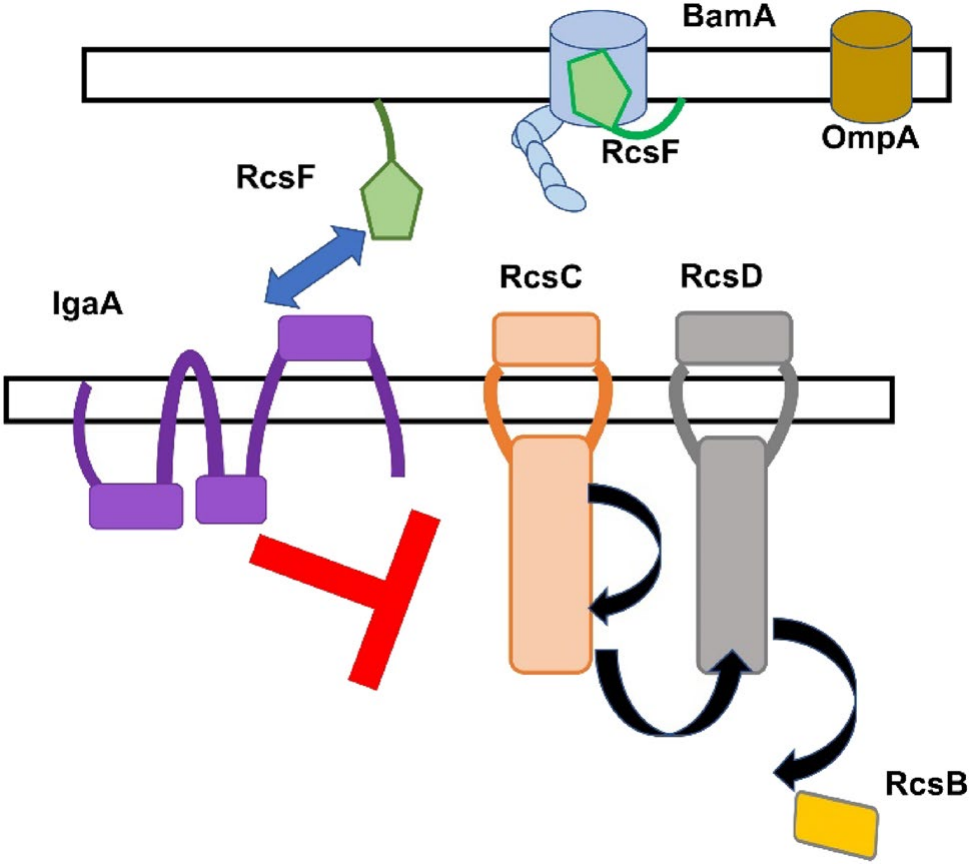
Schematic diagram of RcsF interactions and role in the activation of the Rcs system. In the outer membrane, RcsF interacts with BamA (the essential β- barrel of the BAM machinery, which also mediates RcsF exposure to the cell surface) and OmpA (RcsF-OmpA interaction is not shown) (see text for references). Also, RcsF interacts with the periplasmic domain of the inner membrane protein, IgaA leading to derepression of the Rcs system. The Rcs system is composed of the inner membrane histidine kinase (RcsC) and the cytosolic response regulator (RcsB) in addition the phosphotransfer protein (RcsD) and the negative regulator essential protein (IgaA). Black arrows indicate the direction of the phosphorylation cascade

Within the Rcs system, RcsF senses specific cues, and interact with IgaA, an essential inner membrane protein functioning as the negative regulator of the Rcs system. The interaction between RcsF and IgaA is considered to cause derepression of the Rcs phosphorelay, leading to autophosphorylation of the histidine kinase (RcsC), the transfer of the phosphate group to the phosphotransfer auxiliary protein (RcsD) and finally the phosphorylation of the cytosolic response regulator (RcsB) (Majdalani and Gottesman 2005; Wall et al. 2018 and references therein).(Fig. 1).

In addition, RcsF interacts with BamA (Cho et al. 2014; Konovalova et al. 2014; Rodríguez-Alonso et al. 2020), an essential component of the Bam machinery mediating the insertion of β-barrel proteins in the outer membrane of *E. coli* (Konovalova et al. 2016; Malinverni and Silhavy 2011) and the outer membrane porin OmpA (Konovalova et al. 2014; Cho et al. 2014; Dekoninck et al. 2020).

Based on its important role and key interactions, we reasoned that a peptide interfering with the RcsF function within the Rcs system and/ or interaction might also affect the outer membrane thus affecting bacterial survival. In addition, the crystal structure of RcsF was determined previously as well as its complex with BamA (Leverrier et al. 2011; Rogov et al. 2011; Rodríguez-Alonso et al. 2020). This makes RcsF primary structure a good candidate for derivation of a sequence of antibacterial peptide. An additional advantage is RcsF relatively small molecular mass (12 kDa for the mature protein), so a peptide simulating its structure could potentially overcome the outer membrane barrier.

In this work, we derive the sequence of a novel peptide RcsFmimic (RcsFmim) from RcsF based on the in silico prediction of RcsF- IgaA interaction map. We show that expression of RcsFmim-coding gene causes a constitutive activation of the Rcs system and affects outer membrane permeability resulting in decreased *E. coli* growth. We anticipate that RcsFmim presents a likely candidate for future antibacterial peptide development.

## Materials and methods

### Media, bacterial strains and plasmids

The bacterial strains used in this study are all derivatives of *E. coli* K12 MG1655 carrying a chromosomal *rprA*::*lacZ* fusion at the lambda attachment site (DH300) (Majdalani et al. 2002). This strain was used previously as a reporter strain to monitor the Rcs system activation in the absence and presence of Rcs- specific inducing cues (Cho et al. 2014; Asmar et al. 2017; Hussein et al. 2018; Lach et al. 2023). The *rcsB* and *rcsF* knockout strains were generated by P1 *vir* transduction from the corresponding Keio collection strain (Baba et al. 2006) (obtained from the National Bioresource Project, Japan) to *E. coli* MG1655 DH300 and were checked by corresponding PCR primers complementary to the upstream and downstream genomic locus of each gene.

Unless otherwise stated, LB-Lennox (MP biomedical) was used to culture *E. coli* MG1655 DH300 at 37 °C containing (whenever necessary) the following concentration of antibiotics: chloramphenicol (25 μg/μl), spectinomycin (50 μg/ml) and kanamycin (20 μg/μl). Whenever required, isopropyl β- thiogalactoside (IPTG) was added at a final concentration of 100 µM, L- arabinose and D-fucose at a final concentration of 0.2% weight/ volume.

Plasmids used in this study are all derived from either pSC232 or pNH401 previously described in (Hussein et al. 2018). pSC232 is derived from pSC101 (Dominique and Bouché 1991), modified by inserting *lacIq* and *trc* promoter from pTrc99a plasmid and is a kind gift from Dr Seun-Hyun Cho, Collet lab. pNH401 is derived from the L- arabinose inducible pBAD33 (Guzman et al. 1995) as described in (Hussein et al. 2018). *rcsF*mim nucleotide sequence was synthesized as gene block by IDT (Belgium) and was cloned between NcoI and XbaI restriction sites in pSC232 or SacI and XbaI restriction sites in pNH401. In both cases, the XbaI site was located upstream of an in- frame tag (3X flag tag in pSC232 and 5X His tag in pNH401) followed in both cases by the KpnI restriction site. Standard molecular biology techniques were followed, using Phusion polymerase (Thermo), restriction enzymes (Thermo) and *E. coli* DH5α (Novagen) as cloning strain.

## Prediction of IgaA periplasmic domain three-dimensional structure and RcsF- IgaA interaction:

We constructed a homology model of IgaA periplasmic domain (IgaAperip) (comprised between amino Asp361 to Tyr655) using Phyre2 server (Kelley et al. 2015). The interaction of RcsF (PDB2Y1B) with IgaAperip was predicted using the protein–protein docking protocol of the Molecular Operating Environment (MOE 2019.0102 Chemical Computing Group, Montreal-Canada) (Chemical Computing Group ULC 2023).

For prediction of the three-dimensional structure of RcsFmim, we used wild type RcsF crystal structure (PDB2Y1B) and the amino acids residues 16 to 45 were deleted. In parallel, we built a homology model for RcsFmim using its predicted amino acid sequence and wild type RcsF as a template using Phyre2. The deduced amino acid sequence of RcsFmim was deposited in a patent request to the Egyptian patent office (request number EG/P/2023/1561). RcsFmim- IgaAperip and RcsFmim- BamA interactions were predicted as described above.

Another homology model for IgaAperip was built using AlphaFold2 via ColabFold server and its interaction with RcsFmim was predicted using AlphaFold2-Multimer chimera visual interface (Mirdita et al. 2022).

Predictions using AlphaFold2 were performed by Ann Analysis for Computational Chemistry Academic services (Egypt).

## β-Galactosidase assay

Β- galactosidase activity was measured in exponentially growing *E. coli* MG1655 DH300 transformed with various plasmids as described previously (Miller 1972). In case of measuring Rcs activation in response to stress, mecillinam (amdinocillin) (Sigma-Aldrich, Germany) was added at a final concentration of 0.3 µg/ml when the bacterial OD_600_ reached 0.2 and incubated at 37°C with agitation for one hour before assessing β- galactosidase activity. Briefly, 20 μl of the culture were permeabilized by mixing with 80 μl of permeabilization solution (100 mM Na_2_HPO_4_, 20 mM KCl, 2 mM MgSO_4_, 0.8 mg/ml CTAB (hexadecyltrimethylammonium bromide), 0.4 mg/ml sodium deoxycholate and 5.4 μL/ml β-mercaptoethanol). After one hour incubation at room temperature, 600 μl of substrate solution (60 mM Na_2_HPO_4_, 40 mM NaH_2_PO_4_, 1 mg/ml o-nitrophenyl-β-D-galactoside (ONPG) (Sigma-Aldrich, Germany) and 2.7 μl/ml β-mercaptoethanol were added and the tubes were incubated at 30 ºC until development of the yellow color. The reaction was then stopped by addition of 700 μl of 1 M Na_2_CO_3_ and the absorbance at 420 nm was assessed spectrophotometrically. The Miller units were calculated according to the following equation.

$${\text{Miller units}} = {1}000 \cdot {\text{Abs42}}0{\text{ nm}}/({\text{OD6}}00 \cdot {\text{reaction time}}\left( {{\text{minutes}}} \right) \cdot 0.0{2}\;{\text{ml}}$$

All β- galactosidase assays were repeated at least in three different biological events.

## Assessment of *E. coli* growth and calculation of logarithmic growth rate

To monitor *E. coli* growth, the corresponding strains were cultured overnight in LB-Lennox containing 25 µg/ml chloramphenicol and were grown at 37 °C overnight. On the following day, the cultures were diluted 1/100 in fresh LB-Lennox containing 25 µg/ml chloramphenicol supplemented with either 0.2% L-arabinose or 0.2% D- fucose. The OD_600_ was measured in BioTek plate reader every hour for six hours. The growth rate (k) of the logarithmic phase in liquid cultures was calculated using the exponential equation Nf = N_i_ e^kΔt^. Alternatively, aliquots from the growing cultures were serially diluted and then spotted on LB- agar- chloramphenicol supplemented with either 0.2% L-arabinose or 0.2% L- glucose. Number of Colonies Forming units (CFU/ ml) were calculated and normalized to the CFU/ ml of empty pBAD33/ *E. coli* DH300 or empty pBAD33/ *rcsB*::*kan* tested simultaneously. Each test was repeated at least in three biological replicates.

## Outer membrane integrity/ permeability testing

To test the effect of *rcsF*mim expression of *E. coli* outer membrane integrity, mid- log phase cultures of *E. coli* DH300 or *rcsF* or *rcsB* null mutants transformed with either empty pBAD33, *rcsF*mim-pBAD33 or p*rcsF*wt-pBAD33 were serially diluted and spotted on LB- agar- arabinose plates containing 2% sodium dodecyl sulphate (SDS) or 1 mM ethylene diamine tetra acetic acid disodium salt (EDTA). The plates were incubated overnight at 37 °C. Number of Colonies Forming units (CFU/ ml) were calculated and normalized to the CFU/ ml of empty pBAD33/ *E. coli* DH300 or empty pBAD33/ *rcsB*::*kan* tested simultaneously. Each test was repeated at least in three biological replicates.

To assess the outer membrane permeability of the same strains to the fluorescent probe 8-anilino-1-naphthalenesulfonic acid (ANS) (Loh et al. 1984; Lamers et al. 2013; Schäfer & Wenzel 2020; Tag ElDein et al. 2021), pellets of mid- log phase cultures of *E. coli* DH300 or *rcsF* or *rcsB* null mutants transformed with either empty pBAD33, *rcsF*mim-pBAD33 or p*rcsF*wt-pBAD33 grown in Mueller Hinton broth were washed and resuspended in 5 mM HEPES, and the fluorescent probe ANS (ANS, Sigma-Aldrich, Germany) was added (at a final concentration of 3 mM). Relative fluorescence was calculated as the ratio of ANS fluorescence in the tested strains (normalized to their OD_600_) to the isogenic strain transformed with the empty plasmid. Excitation and emission wavelengths for ANS were 375 and 510 nm, respectively. The assay was performed in independent triplicates with each biological replicate consisting of three technical replicates.

## Figure preparation and statistical analysis

Histograms, violon plots and growth curves represent an average of at least three biological replicates and were prepared using Prism 8 (Graph-Pad Software, Inc.). Statistical analysis was performed using the same software. Statistical significance was calculated based on unpaired t test for Fig. 4a, one way ANOVA (Kruskal–Wallis test) for Fig. 5, multiple t test Fig. 6 and supplementary Figs. 6 and 7 and two way ANOVA for supplementary Fig. 8. In all figures, error bars represent standard error of the means, single asterix denotes P ≤ 0.1, double asterix P ≤ 0.05 and triple asterix P ≤ 0.01.

## Results

## Structural design of RcsF mimic peptide

RcsF- IgaA interaction is a key step in sensing and transducing cell envelope stresses. The periplasmic domain of IgaA (IgaAperip) was shown previously to interact with RcsF in presence of Rcs- inducing cues (Cho et al. 2014; Hussein et al. 2018). The formation of RcsF- IgaA stress- dependent complex is considered a key step in relieving IgaA- mediated repression of the Rcs system. However, the RcsF- IgaAperip complex is not crystallized to date.

Accordingly, a first step in developing an RcsF mimic peptide is determining the important residues required and/ or contributing to IgaA binding.

Since the three-dimensional structure of IgaAperip (extending from Asp361 to Tyr654) is not determined experimentally, we built a homology model for its structure using Phyre2 server (Kelley et al. 2015). We obtained a model with 78.4% confidence that we used for docking to the tertiary structure of RcsF (PDB 2Y1B) using MOE program and protein–protein docking protocol. The tertiary structure of RcsF excludes the first 32 amino acids of the mature amino acids sequence that were shown to be disordered (Leverrier et al. 2011; Rogov et al. 2011). We analyzed the top 15 predicted conformers for RcsF- IgaAperip interaction complex with S score ranging from -30 to -55, indicating high affinity and stability.

**Fig. 2:**
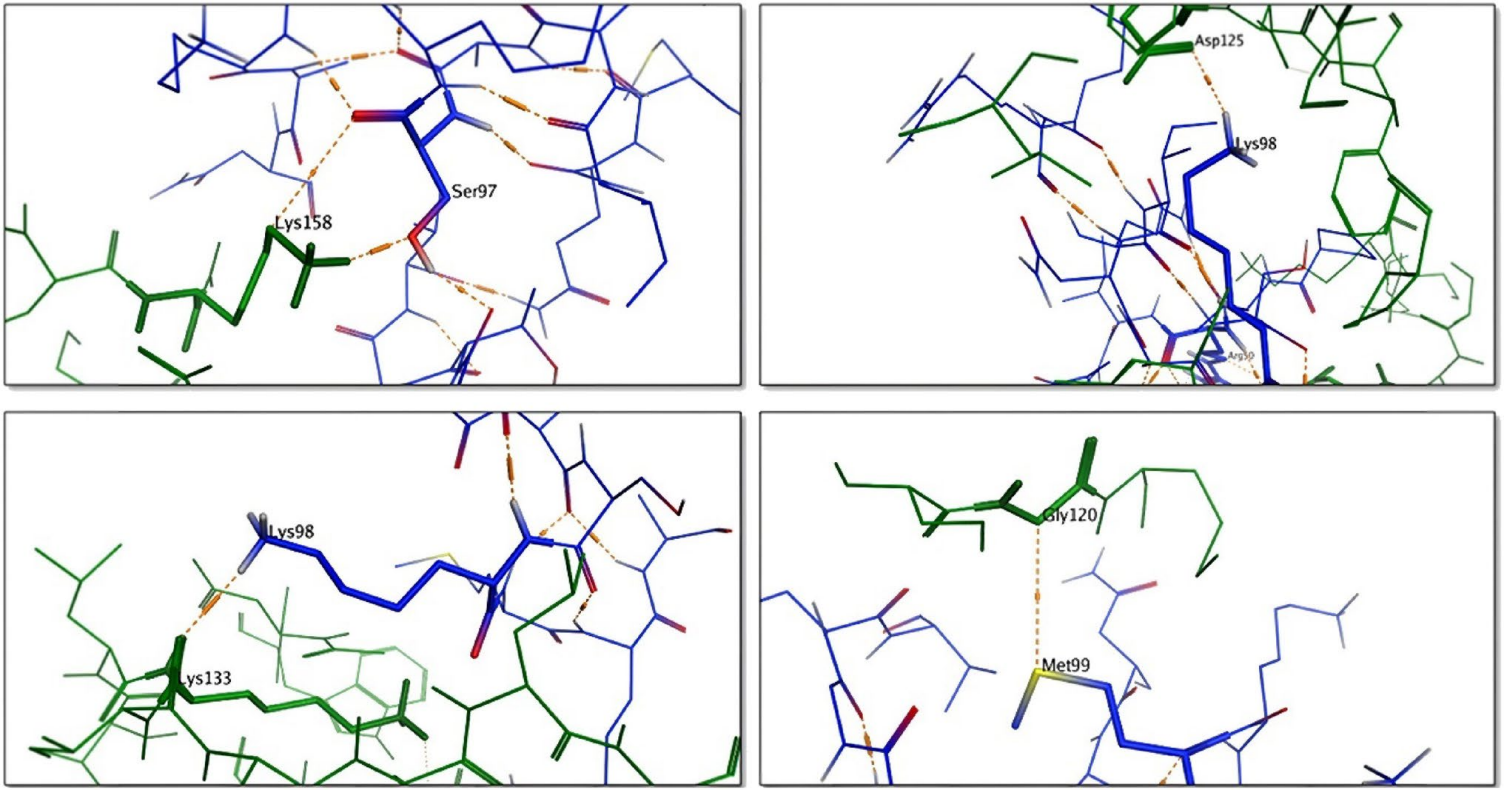
Prediction of RcsF interaction with IgaA periplasmic domain. IgaA periplasmic domain tertiary structure (green) was predicted using Phyre2 with 78.4% confidence and was docked to the tertiary structure of RcsF (PDB 2Y1B) (blue). For convenience, amino acids residues in IgaAperip are numbered from 1 to 295, where Asp1 and Tyr295 corresponds to Asp361 and Tyr654 in the full IgaA ORF

Our docking analysis identified 16 RcsF amino acids residues involved in RcsF- IgaAperip interaction (Fig. 2; Supplementary Table S1). Two residues Ser80 and Asp81 were identified in only two conformers, so we chose to include Ser80. Nine of the identified positions were included between residues Lys90 to Pro116, including residue Lys98 which was identified in 5 different conformers and Ser97 which was identified in 2 conformers. We thought this might represent an indication that the region sandwiched between Lys90 to Pro116 plays a significant role in RcsF- IgaAperip interaction (either structural or functional). Because the role of redox regulation in RcsF stress response was reported but not fully understood to date (Leverrier et al. 2011; Rogov et al. 2008, 2011), we kept 3 out of 4 Cys residues forming the two non- consecutive disulfide bonds. From one side, we preferred to keep Cys109 and Cys124 intact to form the corresponding disulfide bond, since it was shown previously to be important for RcsF stability (Leverrier et al. 2011). On the other hand, we did not include Cys74 to keep Cys118 as a single cysteine that may form a mixed complex with a different protein thus perturbing cell envelope.

**Fig. 3:**
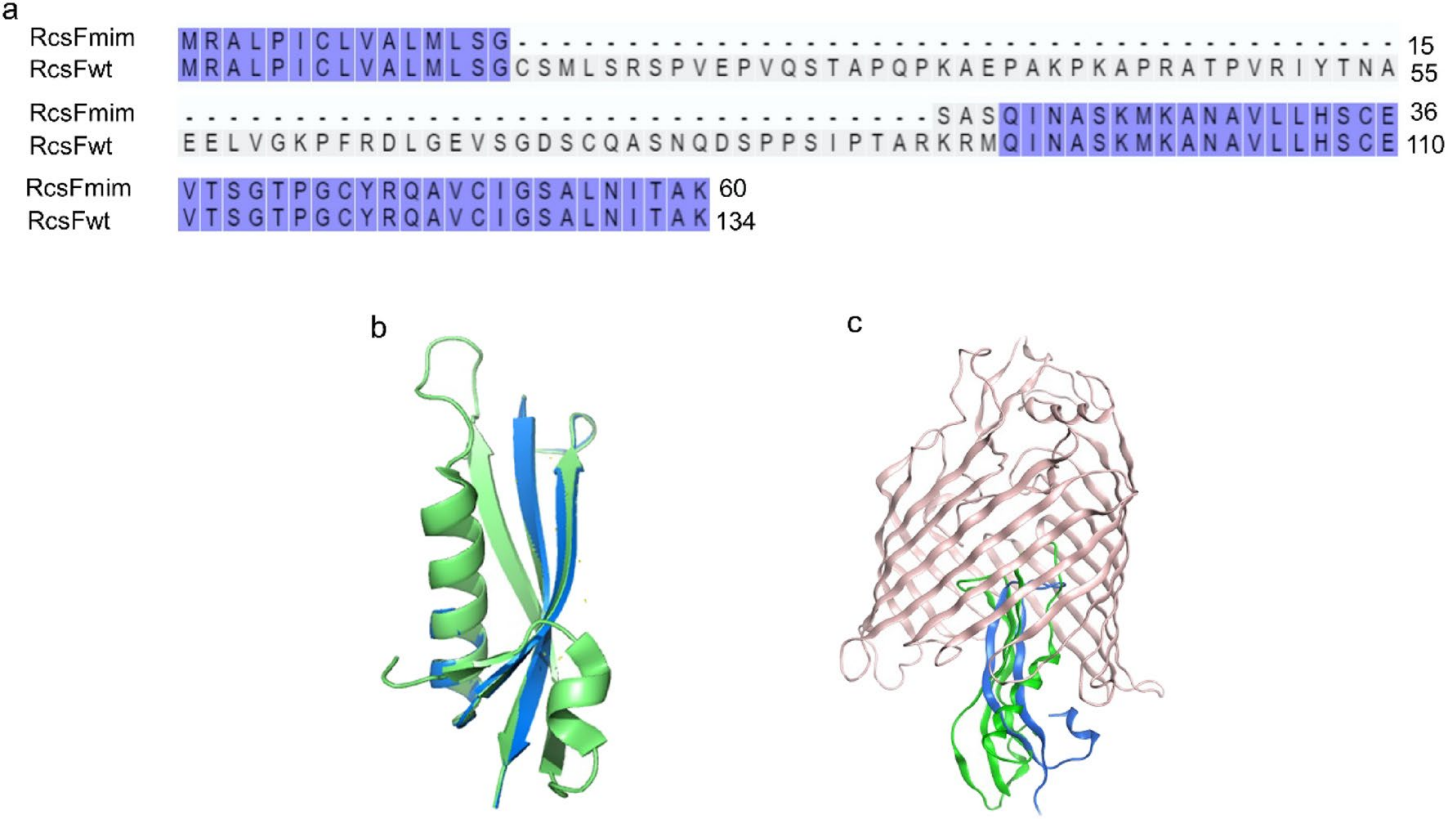
Sequence, predicted tertiary structure and interactions of RcsFmim. **a** Alignment of RcsFmim amino acid sequence with wild type RcsF. Identical amino acids residues are highlighted in blue. **b** Overlay of RcsFmim predicted tertiary structure (blue) with wild type RcsF crystal structure (PDB 2Y1B) (green). **c** Predicted interaction of RcsFmim with BamA. As wild type RcsF (green), RcsFmim (blue) occupies the luminal wall of the BamA β- barrel

Accordingly, the new hypothetical peptide that we named RcsFmimic (RcsFmim) includes the Ser80 and the region extending from Gln93 to Lys134 (Supplementary Table 2, Fig. 3a and b). To confirm the ability of RcsFmim to interact with IgaAperip, the predicted tertiary structure of RcsFmim was docked into the homology model of IgAperip we constructed previously (Supplementary Fig. 1) and showed four amino acids residues likely to be involved in the interaction (Asn28, Met25, Glu36 and Gln37) (corresponding to Asn95, Met99, Glu110 and Gln121 in wild type RcsF, respectively).

To further predict the interaction between RcsFmim and IgaAperip, we constructed a second homology model of IgaAperip and predicted its interaction with RcsFmim using AlphaFold2-Multimer chimera visual interface (Mirdita et al. 2022). RcsFmim residues predicted to interact with IgaAperip were Asn28, Val30, Leu 32, Ser53 and Asn56 (corresponding to Asn102, Val104, Leu106, Ser127 and Asn130 in wild type RcsF) (Supplementary Table 3, Supplementary Fig. 2). Although both prediction methods identified different residues, we reasoned that, taken together, we could assume that RcsFmim will likely interact with IgaAperip.

BamA is the second essential protein that interacts with RcsF. BamA is the key player in insertion of β—barrel proteins into *E. coli* outer membrane. BamA- RcsF interaction mediates RcsF exposure to *E. coli* cell surface and helps RcsF stress sensing function (Konovalova et al. 2014; Cho et al. 2014; Rodríguez-Alonso et al. 2020). Accordingly, we examined if RcsFmim could interact with BamA based on the experimentally determined RcsF- BamA structure (PDB6T1W) (Rodríguez-Alonso et al. 2020). Our docking results predicted that RcsFmim occupies the luminal wall of the BamA β- barrel like wild type RcsF. Similarly, RcsFmim is predicted to form hydrogen bonds with BamA residues Glu470, Gly453, Asp447 and Asn805 (Fig. 2c, Supplementary Fig. 3).

## RcsFmim activates the Rcs system

Based on the predictions described in the previous section, we thought that RcsFmim could positively or negatively affect signaling through the Rcs phosphorelay (either through the possible interaction with IgaAperip or BamA).

Accordingly, we cloned *rcsF*mim sequence in the medium copy number plasmid pSC232 with a C-terminal 3X flag tag. To this end, we mutated the lipobox Cys to Ser, to direct RcsFmim to the periplasm rather than the outer membrane, thus out ruling the rerouting of RcsFmim to the inner membrane (el Rayes et al. 2021). The plasmids were expressed into *E. coli* K-12 MG1655 DH300 carrying a genomic *rprA*-*lacZ* fusion on the chromosome to assess Rcs system activation. *rprA* encodes a small non- coding RNA and its synthesis is controlled by the Rcs system (Majdalani et al. 2002). From now on, *E. coli* K-12 MG1655 DH300 will be designated as wild type strain.

**Fig. 4:**
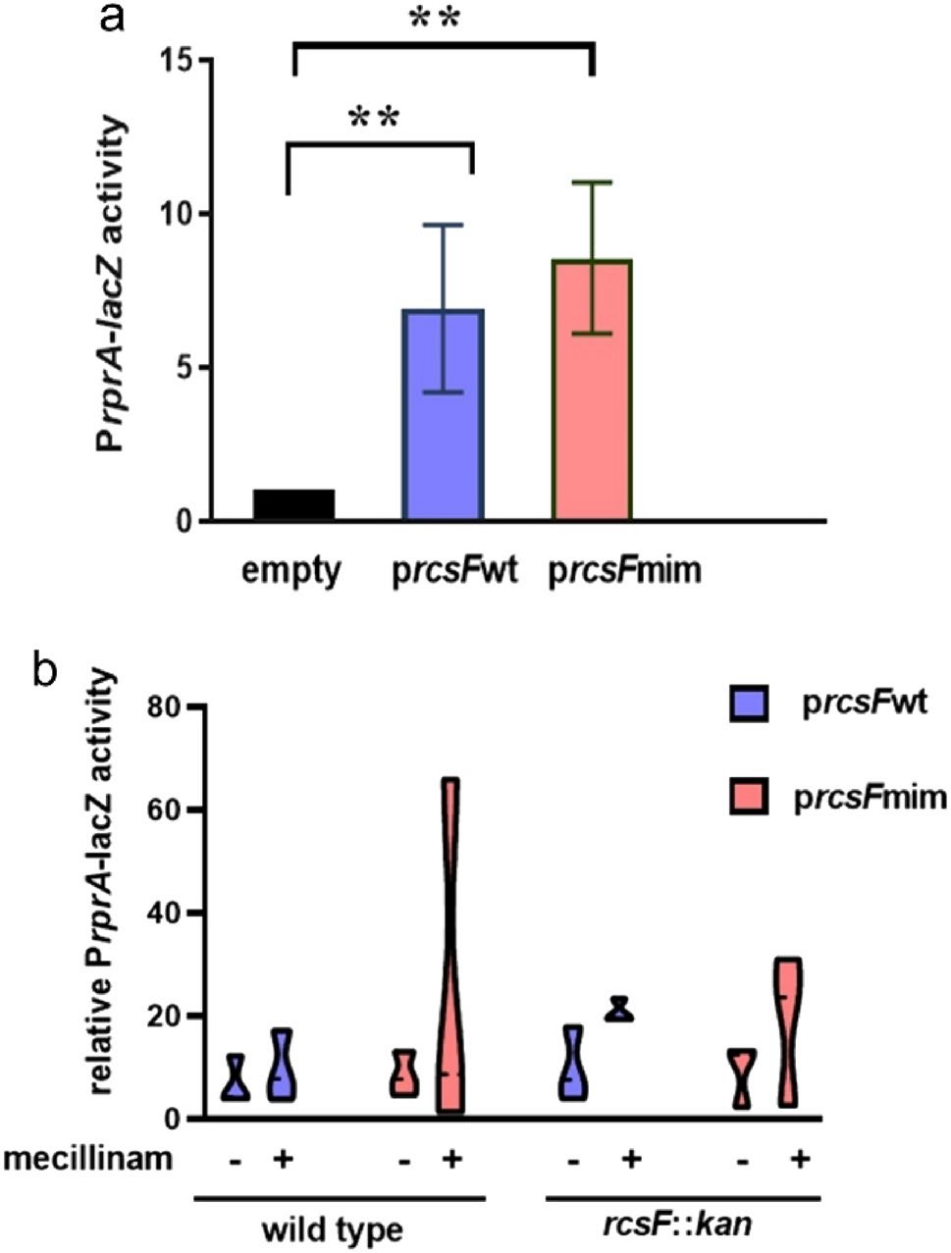
Effect of RcsFmim on Rcs system activity and response to stress. **a** Activation of the Rcs system by expression of *rcsF*mim. Wild type *E. coli* DH300 containing either empty pSC232 or *rcsF*wt-pAM238 or p*rcsF*mim-pSC232 were grown until OD_600_ = 0.6–0.8 and the β-galactosidase activity was measured as previously described. Error bars denote standard error of the mean and double asterix denote statistically significant difference at *P* ≤ 0.05. **b** Effect of RcsFmim on Rcs system response to mecillinam. Wild type or *rcsF* null *E. coli* DH300 containing either *rcsF*wt-pAM238 or p*rcsF*mim-pSC232 were grown until OD_600_ = 0.2 and then treated with mecillinam at a final concentration 0.3 µg/ml. The β-galactosidase activity was measured after one hour as previously described and the activation was calculated relative to the *E. coli* DH300 containing empty pSC232

Induction of expression of *rcsF*mim by adding 100 µM IPTG to the wildtype strain culture (where chromosomal *rcsF* is present) resulted in three-to-four-fold activation of the Rcs system (Fig. 4a). This activation was comparable to the isogenic strain transformed with p*rscF*wt. When we treated the cultures expressing *rcsF*mim with to 0.3 µg/ml mecillinam (amdinocillin), a β- lactam antibiotic known to induce the Rcs system, we observed a variation of Rcs activation over a wide range (Fig. 4b). To further investigate the effect of *rscF*mim independently of chromosomal *rscF*, we transformed p*rcsF*mim-pSC232 in *E. coli* DH300 *rcsF*::*kan* thus testing if *rcsF*mim could relieve IgaA- mediated repression of the Rcs system. Rcs activation in *rscF* null mutant expressing *rcsF*mim in the absence and presence of 0.3 µg/ml of mecillinam were inconsistent, suggesting a possible perturbation of the cell envelope (Fig. 4b).

Taken together, our results suggest that RcsFmim could affect basal Rcs system signaling but might not be sufficient to replace wildtype RcsF.

## RcsFmim slows *E. coli* growth rate predominantly in an Rcs- independent manner

**Fig. 5:**
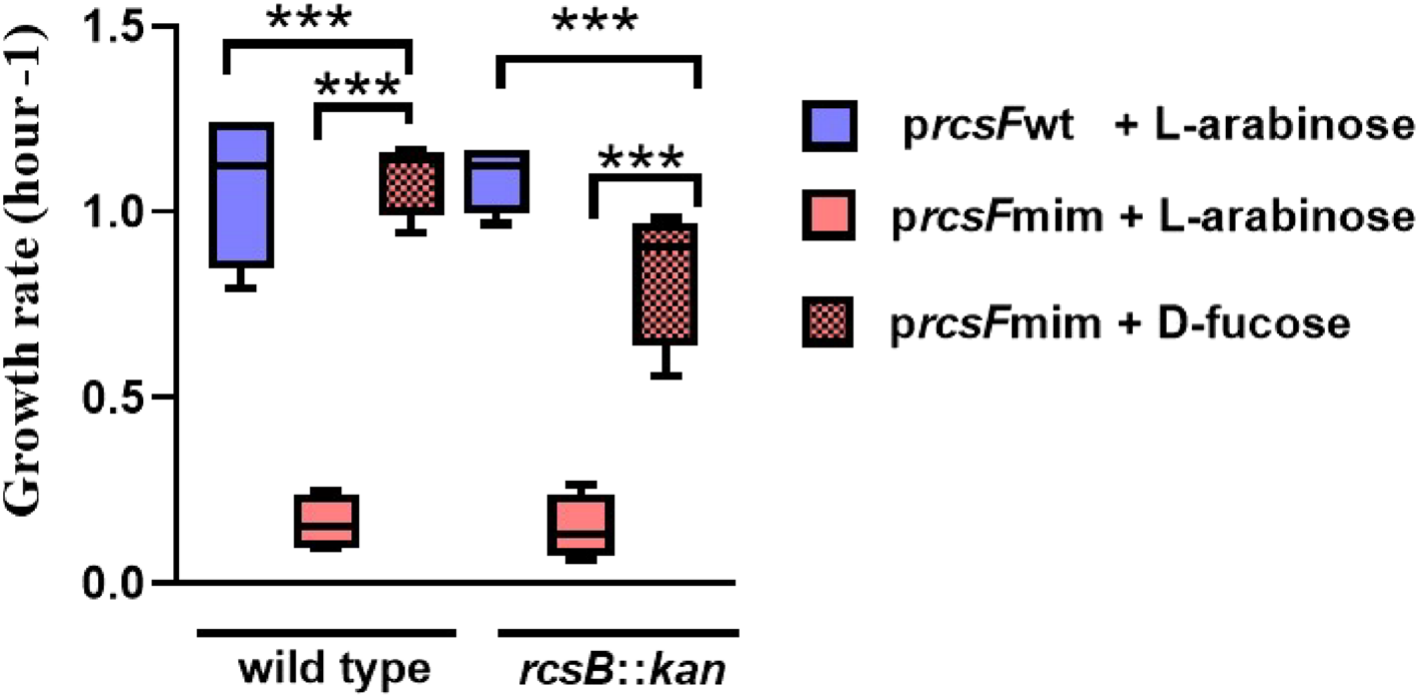
RcsFmim affects *E. coli* growth rate. Overnight cultures of either wild type or *rcsB* null *E. coli* DH300 containing *rscF*wt-pBAD33 or *rcsF*mim-pBAD33 were diluted in fresh LB media containing either 0.2% l-arabinose or 0.2% d-fucose and the OD_600_ was measured each hour for six hours. Triple asterix indicate statistically significant difference *P* ≤ 0.01

As previously mentioned, RcsF interacts with two essential proteins in the outer and inner membrane of *E. coli*, BamA and IgaA, respectively (Konovalova et al. 2014; Cho et al. 2014; Hussein et al. 2018; Rodríguez-Alonso et al. 2020). Based on the predicted IgaAperip- RcsFmim and BamA- RcsFmim interactions, which seem overlapping with wild type RcsF interactions sites, we reasoned that RcsFmim peptide could potentially affect *E. coli* growth. To enable a tighter control of expression of *rcsF*mim, we cloned its sequence in the medium copy number pNH401 (Hussein et al. 2018), derived from pBAD33 (Guzman et al. 1995) under the control the L-arabinose inducible promoter PBAD. First, we confirmed that *rcsF*mim-pBAD33 can induce the Rcs system in the presence of 0.2% L-arabinose (Supplementary Fig. 4). Then, we monitored the growth of *E. coli* DH300 harboring *rcsF*mim-pBAD33 in comparison to the isogenic strains transformed with *rcsF*wt*-*pBAD33 upon induction with 0.2% L- arabinose. We observed that only the strain expressing *rcsF*mim showed severe growth delay (Fig. 5, Supplementary Fig. 5 and 6). To test if this growth delay resulted from the activation of the Rcs system, we transformed *rcsF*mim-pBAD33 and *rcsF*wt*-*pBAD33 in *E. coli* DH300 *rcsB*::*kan* (so turning OFF the Rcs system). To our surprise, RcsFmim resulted also in a significant delay of bacterial growth rate that was only abolished by the addition of D-fucose (a non-metabolizable analogue of L-arabinose that represses expression from the PBAD promoter) (Fig. 5, Supplementary Fig. 7).

Taken together, our results indicate that RcsFmim affects *E. coli* growth predominantly in an Rcs independent manner despite causing Rcs system activation.

## RcsFmim permeabilizes *E. coli* cell envelope

The delayed growth of *E. coli* strains expressing *rcsF*mim independently of Rcs activation and the inconsistency of the complementation experiments, suggested that expression of *rcsF*mim might perturb *E. coli* cell envelope.

Indeed, wild type and *rcsB* null mutants of *E. coli* DH300 transformed with *rcsF*mim-pBAD33 showed increased sensitivity towards 2% SDS and 1 mM EDTA (Supplementary Fig. 8). To confirm further that RcsFmim affects *E. coli* envelope integrity, we assessed the permeability of the outer membrane by measuring the uptake of the fluorescent probe ANS. ANS is a neutral probe that fluoresces upon interaction with hydrophobic ligands or in non-polar environment. Enhanced fluorescence of ANS is an indication of outer membrane perturbation in Gram-negative bacteria (Loh et al. 1984; Lamers et al. 2013; Schäfer & Wenzel 2020; Tag ElDein et al. 2021) Accordingly, we assessed ANS fluorescence of mid- log phase *E. coli* DH300 transformed with either *rcsF*mim-pBAD33 or p*rcsF*wt-pBAD33 compared to the isogenic strain transformed with empty pBAD33.

**Fig. 6:**
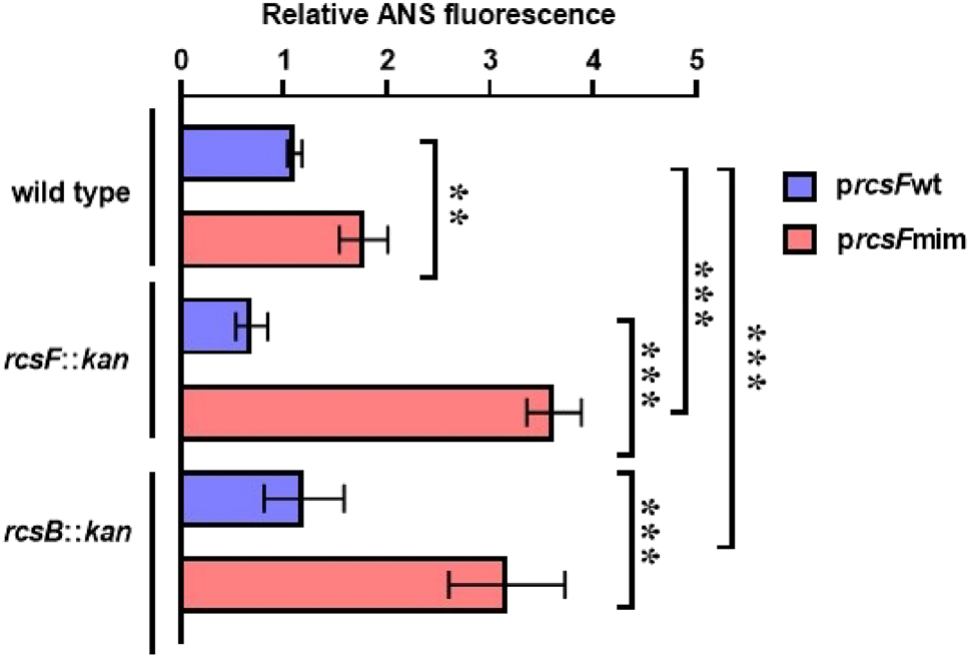
RcsFmim impairs *E. coli* outer membrane. The ANS fluorescence of *E. coli* DH300, *rcsF*::*kan* and *rcsB*::*kan* transformed with either *rcsF*wt-pBAD33 or *rcsF*mim-pBAD33 were calculated relative to that of *E. coli* DH300 containing empty pBAD33. Error bars represent the standard error of the mean, double asterix and triple denote statistical significance *P* < 0.05 and *P* ≤ 0.01, respectively

Only strains transformed with *rcsF*mim-pBAD33 showed enhanced fluorescence, thus indicating outer membrane defect (Fig. 6). We noticed, that expressing *rcsF*mim in the absence of chromosomal *rcsF* (*rcsF*::kan strain) or when the Rcs system is turned OFF (*rcsB*::kan strain), resulted in a higher fluorescence than in the wild type strain (plausible explanations are mentioned in the discussion).

Altogether, our results show that RcsFmim moderately alter Rcs signaling but severely affect bacterial growth through perturbation of outer membrane integrity (Fig. 6).

## Discussion

The problem of antibiotic resistance is a global health challenges threatening humanity, especially in the post-COVID-19 era, as the mortality rates of antimicrobial resistance patients are on the rise (Elmahi et al. 2022; Sulayyim et al. 2022; WHO 2017). Combined efforts are needed to develop non-classically acting antimicrobials. Since Gram-negative cell envelope integrity is a cornerstone in bacterial survival and homeostasis, cell envelope and/ or signaling pathways monitoring its integrity might provide important druggable targets (Hartmann et al. 2010; Bem et al. 2015).

Despite the presence of in-depth studies of bacterial signaling pathways including TCSs, the research area of interfering with signaling pathways sensing and responding to cell envelope challenges via altering protein–protein interaction is largely unexplored although promising (Kahan et al. 2021).

In this work, we suggest a potential antibacterial peptide based on the confirmed but unmapped interaction of RcsF- IgaA. The RcsF-IgaA interaction is reported to increase under Rcs inducing stresses (Cho et al. 2014; Hussein et al. 2018) and to be the first step of activation of the Rcs system. We hypothesized that altering this interaction can potentially compromise this pathway and/ or membrane integrity. Antimicrobial peptides are generally formed of 50–100 amino acids and have a general mechanism of action of disrupting the cell envelope (Barreto-Santamaría et al. 2021; Bechinger & Gorr 2017; Schäfer & Wenzel 2020). Hence, we predicted that interfering with RcsF-IgaA interaction will lead to a sequence for a probable new antimicrobial peptide affecting signaling through the Rcs system and/ or affecting cell envelope integrity.

We based our reasoning that Rcs is a complex phosphorelay, with three auxiliary proteins (RcsF, IgaA and RcsD). One main advantage of altering signaling through the Rcs system is its highly conserved nature in various Enterobacterial genera (Wall, et al. 2018; Majdalani & Gottesman 2005; Meng et al. 2021). Accordingly, an Rcs-acting peptide may be efficient against *E. coli*, *Salmonella* spp, *Klebsiella* sp and *Enterobacter* spp.

Based on previous work on RcsF structure (Leverrier et al. 2011; Rogov et al. 2011) and our docking analysis, we predicted 16 amino acids residues to be important for RcsF-IgaA interaction and/ or RcsF signaling role. We included those 16 amino acid residues in a hypothetical peptide, RcsFmim, and predicted its interaction with IgaA.

Prior to submission of this work, Lach and co-workers published the results of three independent genetic screens which identified, among others, RcsF mutants defective in signaling to IgaA. Some of the RcsF residues identified in their study were included between Gln93 to Lys134, identified through our in silico approach and included in RcsFmim (Lach et al. 2023).

Previous work by Rodriguez- Alonso showed that interaction of wild type RcsF with BamA β-barrel could be divided into three zones, Zone 1 (Z1) including Arg488, Asn643, Tyr 465 and Gln466, Zone 2 (Z2) including Arg592 and Arg634 and Zone 3 (Z3) which is one of the components of the proposed lateral gate of the β barrel (Rodríguez-Alonso et al. 2020). Computational docking of RcsFmim with BamA revealed that RcsFmim occupied the interaction region in BamA and established some hydrogen bonds similar to wild type RcsF in addition to extra hydrogen bonding with other amino acids in the interaction zones (Arg391, Asn459, Arg 526, Glu470, Gly486 and Pro782). Hence, we hypothesized that RcsFmim might interact with BamA or compete with the wildtype RcsF causing an envelope-disrupting phenotype. Both this assumption, structure of RcsFmim and its conformational changes upon binding to partners are yet to be confirmed experimentally.

In addition, we demonstrate that expression of *rscFmim* resulted in increased membrane permeability and a significant delay in *E. coli* cell growth. This growth delay seems to be related to a general stress on the cell envelope rather than the specific activation of the Rcs system for three reasons. First, the growth defect of *E. coli* containing a medium copy number plasmid with *rcsF*mim is not abolished when the Rcs system is turned OFF (*rcsB* null mutants). Second, normal growth is restored only in presence of D-fucose whether the Rcs system is functional or not. Third, *E. coli* expressing *rcsF*mim exhibited increased permeability to ANS indicating outer membrane defect (Fig. 6). The permeability increased in *rcsF* null mutant, probably due to the absence of adequate interaction with wild type RcsF protein partners, leaving room for only aberrant interactions with RcsFmim. Similarly, we observed an increased fluorescence in *rcsB::kan* strain compared to wild type strain when both contained *rcsFmim*-pBAD33, which may outline a limited role of a functional Rcs system in balancing the perturbation caused by RcsFmim.

Interestingly, *rcsF*mim does not hinder chromosomal RcsF interaction with IgaA, as demonstrated by the ability of the wildtype strain transformed with p*rscF*mim-pBAD33 to respond to mecillinam. However, we could not conclude whether *rscF*mim is sufficient to complement the *rscF* null mutant.

The specific in situ target(s) of RcsFmim, is/are yet to be explored. It is noteworthy to mention that RcsF interacts with the outer membrane porin OmpA, which also plays an important role in preserving the outer membrane integrity (Konovalova et al. 2014; Cho et al. 2014; Dekoninck et al. 2020). Whether the effect of RcsFmim is mediated through an aberrant interaction or a stalled complex formation with one or more of the studied proteins (IgaA or BamA, or OmpA) is still under investigation.

Overall, we suggest the sequence of the new peptide RcsFmim, that may serve as an interesting research tool to study cell envelope perturbation and the Rcs system signaling. Future studies are also needed to test whether the purified RscFmim can be uptake by intact *E. coli*, causing a bacteriostatic or bactericidal effect.

### Supplementary Information

Below is the link to the electronic supplementary material.

Supplementary file1 (DOCX 975 KB)

Supplementary file2 (DOCX 21 KB)

## References

[CR1] Asmar AT, Ferreira JL, Cohen EJ, Cho S-H, Beeby M, Hughes KT, Collet J-F (2017). Communication across the bacterial cell envelope depends on the size of the periplasm. PLoS Biol.

[CR2] Baba T, Ara T, Hasegawa M, Takai Y, Okumura Y, Baba M, Datsenko KA, Tomita M, Wanner BL, Mori H (2006). Construction of Escherichia coli K-12 in-frame, single-gene knockout mutants: the Keio collection. Mol Syst Biol.

[CR3] Barreto-Santamaría A, Arévalo-Pinzón G, Patarroyo MA, Patarroyo ME (2021). How to combat gram-negative bacteria using antimicrobial peptides: a challenge or an unattainable goal?. Antibiotics.

[CR4] Bechinger B, Gorr S (2017). Antimicrobial peptides: mechanisms of action and resistance. J Dental Res.

[CR5] Bem AE, Velikova N, Pellicer MT, van Baarlen P, Marina A, Wells JM (2015). Bacterial histidine kinases as novel antibacterial drug targets. ACS Chem Biol.

[CR6] Chemical Computing Group ULC (2023) Molecular Operating Environment (MOE) 2022.02

[CR7] Cho SH, Szewczyk J, Pesavento C, Zietek M, Banzhaf M, Roszczenko P, Asmar A, Laloux G, Hov AK, Leverrier P, van der Henst C, Vertommen D, Typas A, Collet JF (2014). Detecting envelope stress by monitoring β-barrel assembly. Cell.

[CR8] Dekoninck K, Létoquart J, Laguri C, Demange P, Bevernaegie R, Simorre JP, Dehu O, Iorga BI, Elias B, Cho SH, Collet JF (2020). Defining the function of OmpA in the Rcs stress response. Elife.

[CR9] Delhaye A, Collet JF, Laloux G (2016). Fine-tuning of the Cpx envelope stress response is required for cell wall homeostasis in Escherichia coli. Mbio.

[CR10] Dominique G, Jean-Pierre B (1991). No TitColE1-type vectors with fully repressible replicational. Gene.

[CR11] El Rayes J, Szewczyk J, Deghelt M, Csoma N, Matagne A, Iorga BI, Cho SH, Collet JF (2021). Disorder is a critical component of lipoprotein sorting in Gram-negative bacteria. Nat Chem Biol.

[CR12] Elmahi OKO, Uakkas S, Olalekan BY, Damilola IA, Adedeji OJ, Hasan MM, dos Santos Costa AC, Ahmad S, Essar MY, Thomson DJ (2022). Antimicrobial resistance and one health in the post COVID-19 era: what should health students learn?. Antimicrob Resist Infect Control.

[CR13] Guzman L-M, Belin D, Carson MJ, Beckwith J (1995). Tight regulation, modulation, and high-level expression by vectors containing the arabinose P BAD promoter. J Bacteriol.

[CR14] Hartmann M, Berditsch M, Hawecker J, Ardakani MF, Gerthsen D, Ulrich AS (2010). Damage of the bacterial cell envelope by antimicrobial peptides gramicidin S and PGLa as revealed by transmission and scanning electron microscopy. Antimicrob Agents Chemother.

[CR15] Hussein N, Cho S-H, Laloux G, Siam R, Collet J-F (2018). Distinct domains of Escherichia coli IgaA connect envelope stress sensing and down- regulation of the Rcs phosphorelay across subcellular compartments. PLoS Genet.

[CR16] Kahan R, Worm DJ, de Castro GV, Ng S, Barnard A (2021). Modulators of protein-protein interactions as antimicrobial agents. RSC Chem Biol.

[CR17] Kelley LA, Mezulis S, Yates CM, Wass MN, Sternberg MJE (2015). The Phyre2 web portal for protein modeling, prediction and analysis. Nat Protoc.

[CR18] Konovalova A, Perlman DH, Cowles CE, Silhavy TJ (2014). Transmembrane domain of surface-exposed outer membrane lipoprotein RcsF is threaded through the lumen of -barrel proteins. Proc Natl Acad Sci.

[CR19] Konovalova A, Mitchell AM, Silhavy TJ (2016). A lipoprotein/b-barrel complex monitors lipopolysaccharide integrity transducing information across the outer membrane. Elife.

[CR20] Lach SR, Kumar S, Kim S, Im W, Konovalova A (2023). Conformational rearrangements in the sensory RcsF/OMP complex mediate signal transduction across the bacterial cell envelope. PLoS Genet.

[CR21] Lamers RP, Cavallari JF, Burrows LL (2013). The efflux inhibitor phenylalanine-arginine beta-naphthylamide (PAβN) permeabilizes the outer membrane of gram-negative bacteria. PLoS ONE.

[CR22] Leblanc SKD, Oates CW, Raivio TL (2011). Characterization of the induction and cellular role of the BaeSR two-component envelope stress response of *Escherichia coli*. J Bacteriol.

[CR23] Leverrier P, Declercq JP, Denoncin K, Vertommen D, Hiniker A, Cho SH, Collet JF (2011). Crystal structure of the outer membrane protein RcsF, a new substrate for the periplasmic protein-disulfide isomerase DsbC. J Biol Chem.

[CR24] Loh B, Grant C, Hancock REW (1984). Use of the fluorescent probe 1-*N*-phenylnaphthylamine to study the interactions of aminoglycoside antibiotics with the outer membrane of *Pseudomonas aeruginosa*. Antimicrob Agents Chemother.

[CR25] Majdalani N, Gottesman S (2005). THE RCS PHOSPHORELAY: a complex signal transduction system. Annu Rev Microbiol.

[CR26] Majdalani N, Hernandez D, Gottesman S (2002). Regulation and mode of action of the second small RNA activator of RpoS translation, RprA. Mol Microbiol.

[CR27] Malinverni JC, Silhavy T (2011). Assembly of outer membrane β-barrel proteins: the bam complex. EcoSal plus.

[CR28] Meng J, Young G, Chen J (2021). The Rcs system in Enterobacteriaceae: envelope stress responses and virulence regulation. Front Microbiol.

[CR29] Miller J (1972). Experiments in molecular genetics.

[CR30] Mirdita M, Schütze K, Moriwaki Y, Heo L, Ovchinnikov S, Steinegger M (2022). ColabFold: making protein folding accessible to all. Nat Methods.

[CR31] Rodríguez-Alonso R, Létoquart J, Nguyen VS, Louis G, Calabrese AN, Iorga BI, Radford SE, Cho SH, Remaut H, Collet JF (2020). Structural insight into the formation of lipoprotein-β-barrel complexes. Nat Chem Biol.

[CR32] Rogov VV, Schmöe K, Löhr F, Rogova NYu, Bernhard F, Dötsch V (2008). Modulation of the Rcs-mediated signal transfer by conformational flexibility. Biochem Soc Trans.

[CR33] Rogov VV, Rogova NY, Bernhard F, Löhr F, Dötsch V (2011). A disulfide bridge network within the soluble periplasmic domain determines structure and function of the outer membrane protein RCSF. J Biol Chem.

[CR34] Schäfer AB, Wenzel M (2020). A how-to guide for mode of action analysis of antimicrobial peptides. Front Cell Infect Microbiol.

[CR35] Silhavy T, Kahne D, Walker S (2010). The bacterial cell envelope. Cold Spring Harb Perspect Biol.

[CR36] Stout V, Gottesman S (1990). RcsB and RcsC: a two-component regulator of capsule synthesis in *Escherichia coli*. J Bacteriol.

[CR37] Sulayyim HJA, Ismail R, Hamid AA, Ghafar NA (2022). Antibiotic resistance during COVID-19: a systematic review. Int J Environ Res Public Health.

[CR38] Tag ElDein MA, Yassin AS, El-Tayeb O, Kashef MT (2021). Chlorhexidine leads to the evolution of antibiotic-resistant Pseudomonas aeruginosa. Eur J Clin Microbiol Infect Dis.

[CR39] Wall E, Majdalani N, Gottesman S (2018). The complex Rcs regulatory cascade. Annu Rev Microbiol.

[CR40] WHO (2017) No Title. http://www.emro.who.int/egy/egypt-events/antimicrobial-resistance-national-action-plan.html. Accessed 29 Feb 2020

