# Supplementary material for: Altering Escherichia coli envelope integrity by mimicking the lipoprotein RcsF
Source: Arch Microbiol. 2023 Dec 9;206(1):12. doi: 10.1007/s00203-023-03733-3 (PMC10710380; doi:10.1007/s00203-023-03733-3)
Supplement: Supplementary file 1 — Supplementary file1 (DOCX 975 KB) [file 203_2023_3733_MOESM1_ESM.docx]

Journal: Archives of Microbiology

Manuscript Title: Altering *Escherichia coli* Envelope Integrity by Mimicking the Lipoprotein RcsF.

Author name:

Moustafa A. Tag ElDein^1^*, Noha G. Mohamed^2^*, Yasser E. Shahein^3^, Laila Ziko^4^ and Nahla A. Hussein§^3^

* These authors contributed equally.

§ corresponding author

^1^Microbiology and Immunology Department, Faculty of Pharmacy, Cairo University, Egypt.

^2^Pharmaceutical Chemistry Department, Faculty of Pharmacy, Sphinx University, Assiut, Egypt.

^3^ Molecular Biology Department, Biotechnology Research Institute, National Research Centre, Cairo, Egypt.

^4^ Department of Biochemistry, School of Life and Medical Sciences, University of Hertfordshire hosted by the Global Academic Foundation, New Administrative Capital, Cairo, Egypt.

Corresponding author

**Nahla A. Hussein**

Molecular Biology Department, Biotechnology Research Institute, National Research Centre, Cairo, Egypt.

Nahlahussein@aucegypt.edu, ORCID 0000-0003-2520-0896


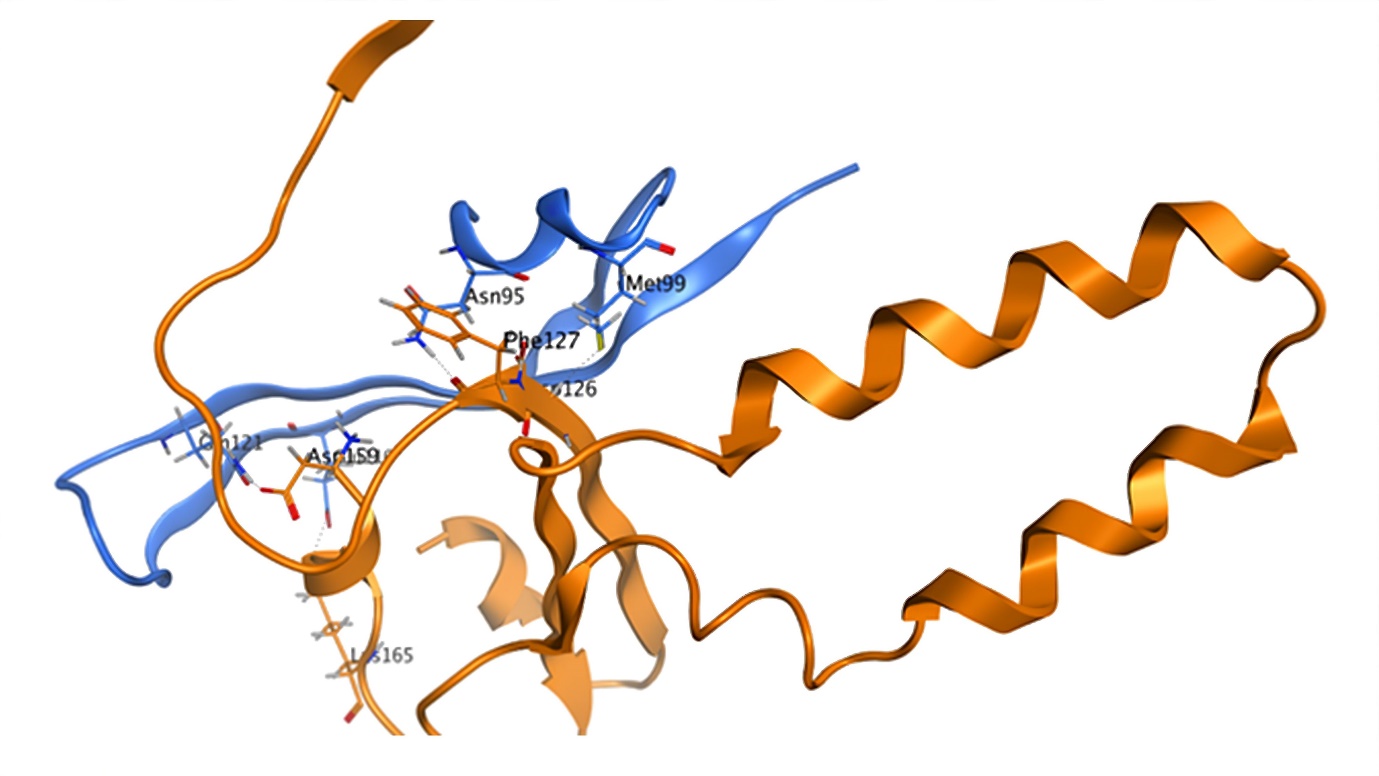


Supplementary Fig. 1

Predicted interaction of RcsFmim with IgaAperip using Phyre2. RcsFmim is shown in blue and IgaAperip in orange.

For convenience, amino acids residues in IgaAperip are numbered from 1 to 295, where Asp1 and Tyr295. In RcsFmim, amino acids residues are numbered based on wild type RcsF sequence.


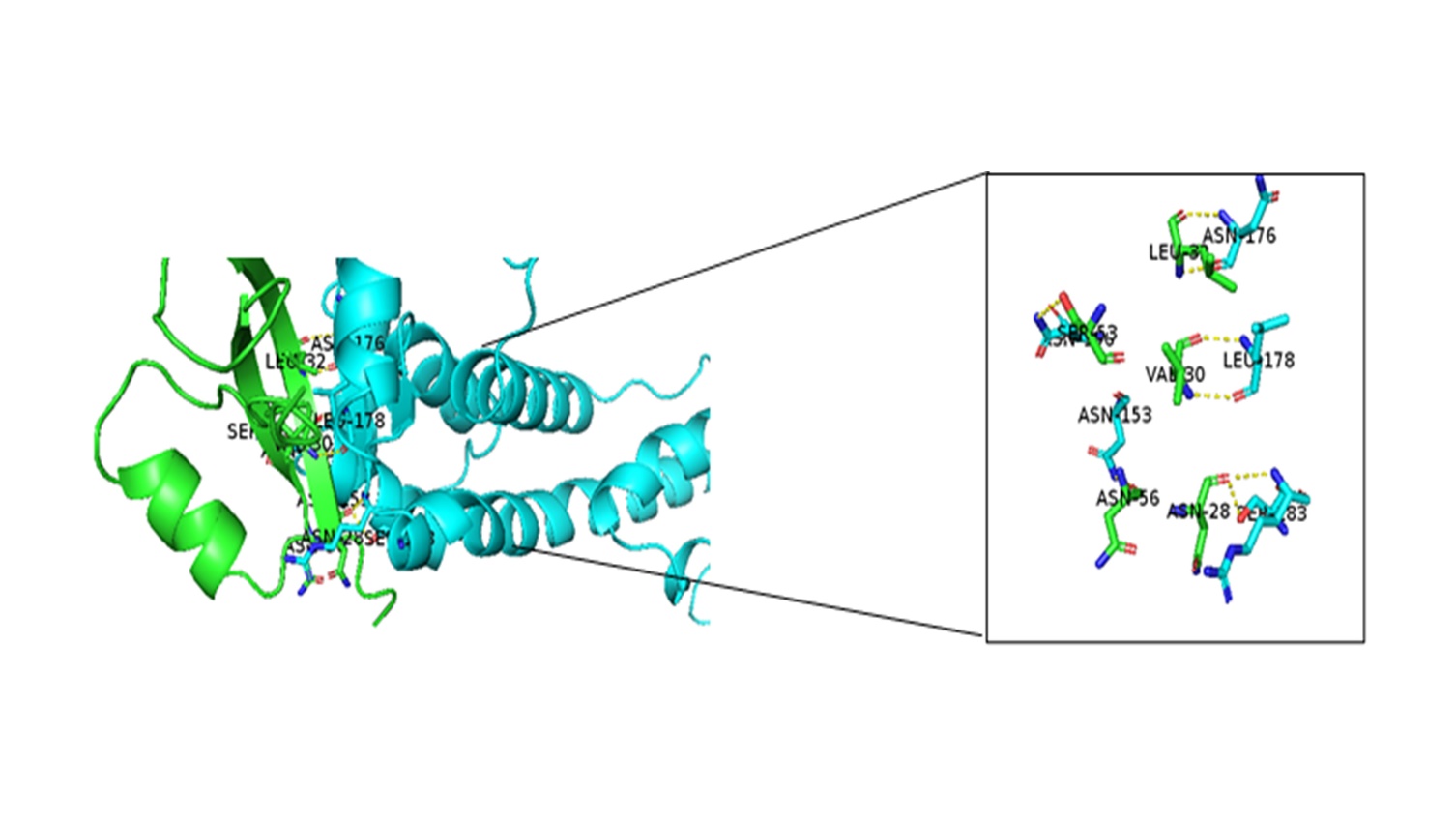


Supplementary Fig. 2

Predicted interaction of RcsFmim with IgaAperip using AlphaFold2 and visulaize by pymol. RcsFmim is shown in green and IgaAperip in blue. (please refer to Supplementary table 2).


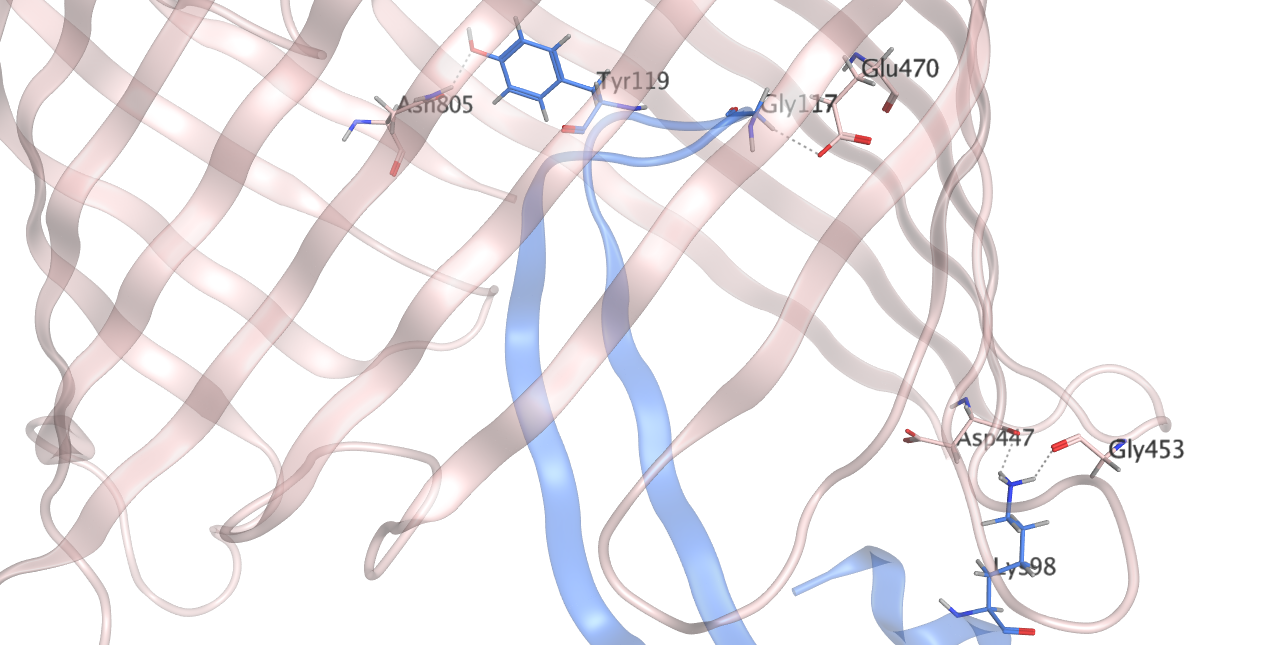


Supplementary Fig. 3

Predicted interaction of RcsFmim with BamA. RcsFmim is shown in blue. RcsFmim is predicted to form hydrogen bonds with BamA residues Glu470, Gly453, Asp447 and Asn805


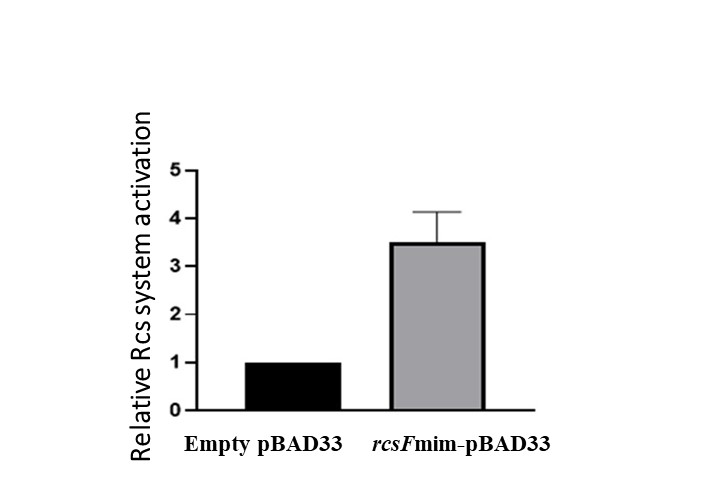


***

Supplementary Fig. 4

Activation of the Rcs system by *rcsFmim*-pBAD33. Wild type *E. coli* DH300 containing either empty pBAD33 or p*rcsF*mim-pBAD33 - were grown until OD600 0.6-0.8 and then the β- galactosidase activity was measured as previously described. The experiment was done in three different biological events. Error bars denote standard error of the mean and triple asterix denote statistically significant difference at P ≤0.01


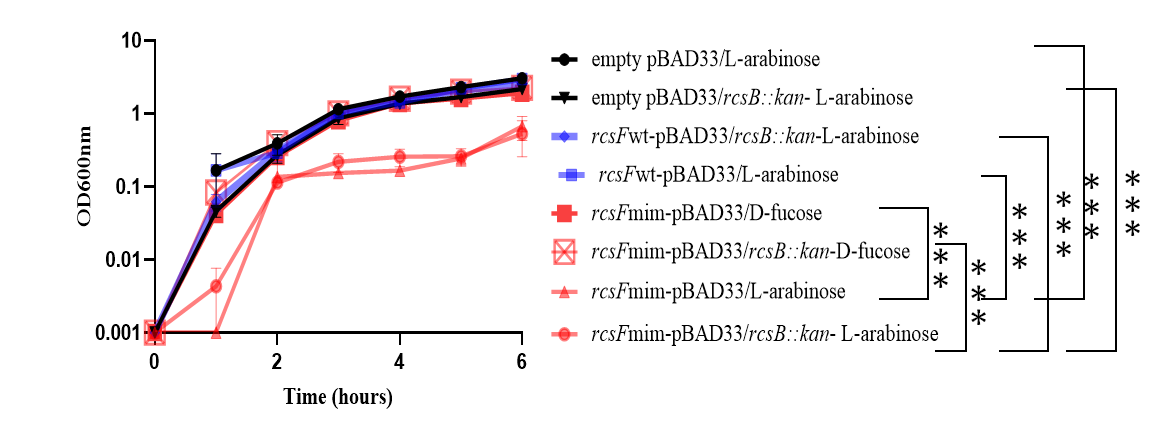


Supplementary Fig. 5 Effect of RcsFmim on *E. coli* growth. Overnight cultures of either wild type or *rcsB* null *E. coli* DH300 containing either empty pBAD33 or *rcsF*mim-pBAD33 or *rscF*wt-pBAD33 were diluted in fresh LB media containing either 0.2% L-arabinose or 0.2% D-fucose and the OD600 was measured each hour for six hours. Growth monitoring was done in three biological events. Error bars denote standard error of the mean. Multiple t- test was used to test statistical significance, triple asterix denotes P ≤0.01.


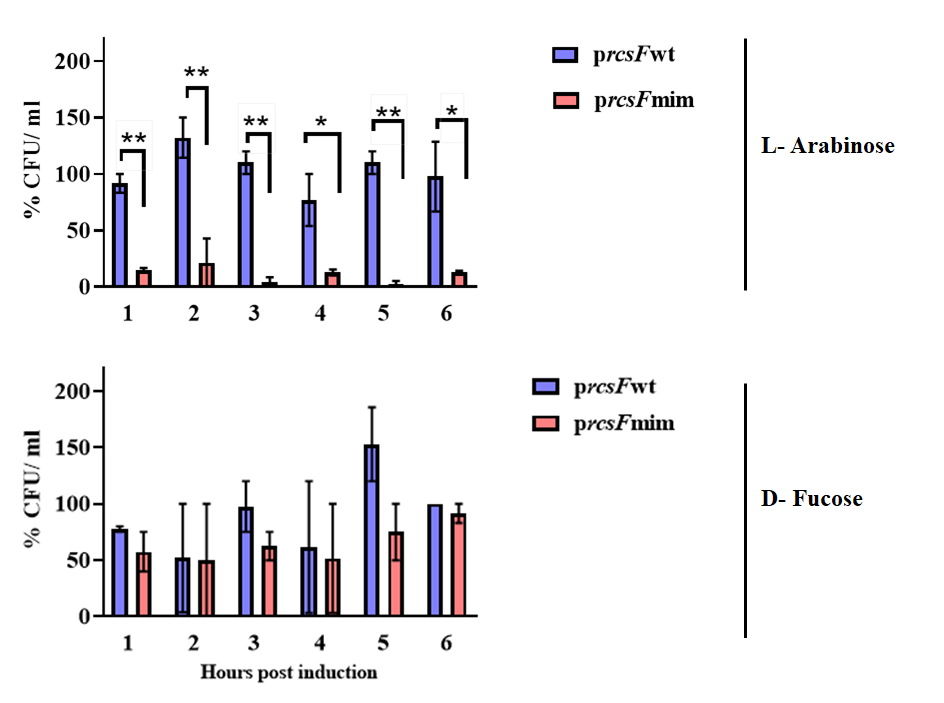


Supplementary Fig. 6 Effect of RcsFmim on *E. coli* growth. Overnight cultures of wild type *E. coli* DH300 containing either empty pBAD33 or *rcsF*mim-pBAD33 or *rscF*wt-pBAD33 were diluted in fresh LB media containing either 0.2% L-arabinose or 0.2% D-fucose and, aliquots from the growing cultures were serially diluted and then spotted on LB‑ agar- chloramphenicol supplemented with either 0.2% L-arabinose or 0.2% L‑ glucose. Number of Colonies Forming units (CFU/ ml) were calculated and normalized to the CFU/ ml of empty pBAD33/ *E. coli* DH300 tested simultaneously.

In the presence of the inducer (L‑ arabinose), survival of the strain transformed with *rcsFmim*‑ pBAD33 is remarkably less than the isogenic strains transformed with *rcsF*wt-pBAD33. In the presence of D‑ fucose, we observed no statistically significant differences between survival of *rcsFmim*‑ pBAD33/ *E. coli* DH300 and *rcsF*wt-pBAD33 / *E. coli* DH300.

Each test was repeated in three biological replicates and statistical significance determined using multiple t test. Error bars represent standard error of the means, single asterix denotes P ≤0.1 and double asterix denotes P ≤0.05.


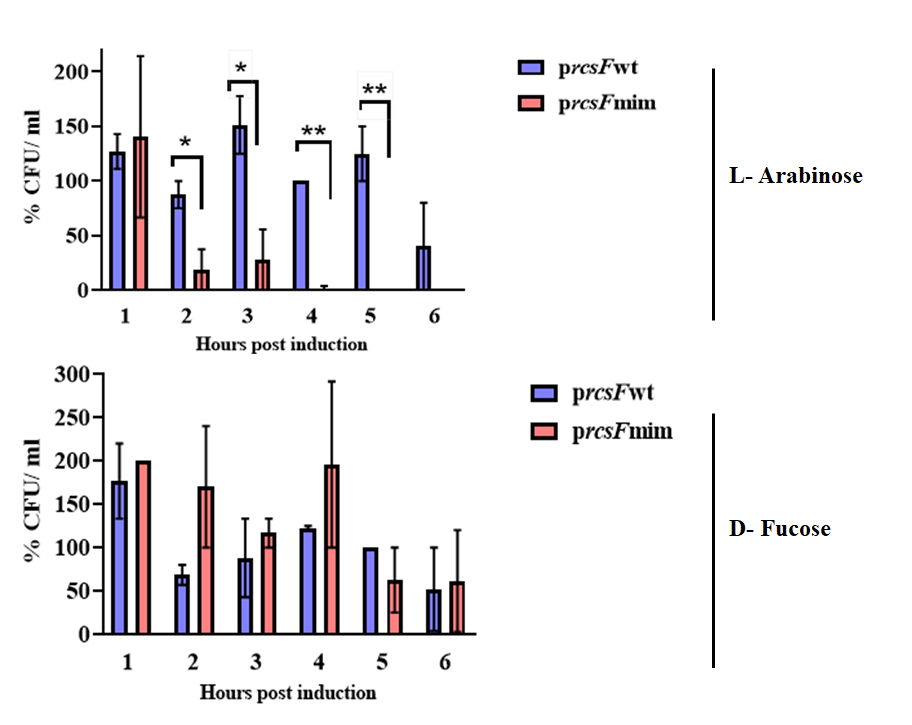


Supplementary Fig. 7 Effect of RcsFmim on *E. coli* growth. Overnight cultures of *rcsB* null mutant *E. coli* DH300 containing either empty pBAD33 or *rcsF*mim-pBAD33 or *rscF*wt-pBAD33 were diluted in fresh LB media containing either 0.2% L-arabinose or 0.2% D-fucose and , aliquots from the growing cultures were serially diluted and then spotted on LB‑ agar- chloramphenicol supplemented with either 0.2% L-arabinose or 0.2% L‑ glucose. Data was calculated as outlined in Supplementary Fig. 6. In the presence of the inducer (L‑ arabinose), survival of the strain transformed with *rcsFmim*‑ pBAD33 is remarkably less than the isogenic strains transformed with *rcsF*wt-pBAD33 (except at one and six hours). Since in this strain the Rcs system is turned OFF, the effect of RcsFmim on *E. coli* growth is predominantly independent on the Rcs system.

Each test was repeated in three biological replicates and statistical significance determined using multiple t test. Error bars represent standard error of the means, single asterix denotes P ≤0.1 and double asterix denotes P ≤0.05.


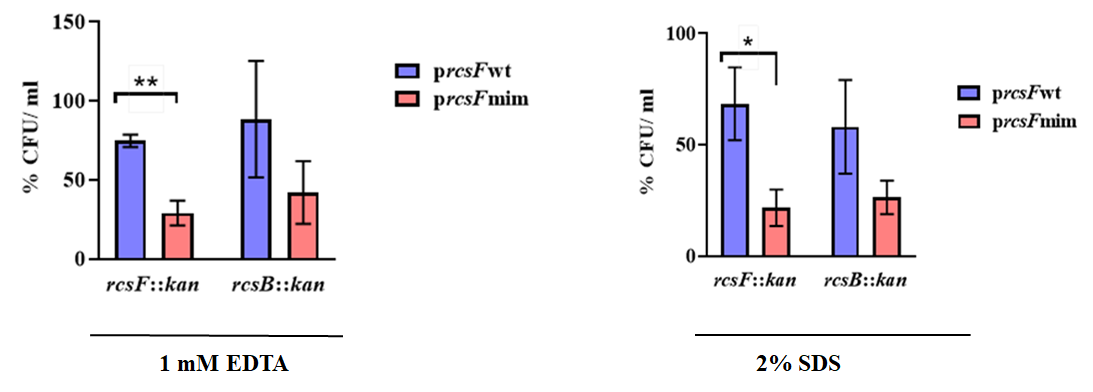


Figure 8: Effect of RcsFmim on *E. coli* sensitivity to EDTA and SDS. Mid- log phase cultures of *E. coli* DH300 *rcsF* or *rcsB* null mutants transformed with either empty pBAD33, *rcsF*mim-pBAD33 or p*rcsF*wt‑pBAD33 were serially diluted and spotted on LB- agar plates containing either 1 mM ethylene diamine tetra acetic acid disodium salt (EDTA) (left panel) or 2% sodium dodecyl sulphate (SDS) (right panel). *rcsFmim*‑ pBAD33/ *E. coli* shows increased sensitivity to both 2% SDS and 1 mM EDTA in *rcsF*::kan strain. Calculation and normalization were done as described in Supplementary Figure 6. Each test was repeated in three biological replicates and statistical significance determined using two- way ANOVA. Error bars represent standard error of the means, single asterix denotes P ≤0.1 and double asterix denotes P ≤0.05.
