# Supplementary material for: Altering Escherichia coli envelope integrity by mimicking the lipoprotein RcsF
Source: Arch Microbiol. 2023 Dec 9;206(1):12. doi: 10.1007/s00203-023-03733-3 (PMC10710380; doi:10.1007/s00203-023-03733-3)
Supplement: Supplementary file 2 — Supplementary file2 (DOCX 21 KB) [file 203_2023_3733_MOESM2_ESM.docx]

Journal: Archives of Microbiology

Manuscript Title: Altering *Escherichia coli* Envelope Integrity by Mimicking the Lipoprotein RcsF.

Author name:

Moustafa A. Tag ElDein^1^*, Noha G. Mohamed^2^*, Yasser E. Shahein^3^, Laila Ziko^4^ and Nahla A. Hussein§^3^

* These authors contributed equally.

§ corresponding author

^1^Microbiology and Immunology Department, Faculty of Pharmacy, Cairo University, Egypt.

^2^Pharmaceutical Chemistry Department, Faculty of Pharmacy, Sphinx University, Assiut, Egypt.

^3^ Molecular Biology Department, Biotechnology Research Institute, National Research Centre, Cairo, Egypt.

^4^ Department of Biochemistry, School of Life and Medical Sciences, University of Hertfordshire hosted by the Global Academic Foundation, New Administrative Capital, Cairo, Egypt.

Corresponding author

**Nahla A. Hussein**

Molecular Biology Department, Biotechnology Research Institute, National Research Centre, Cairo, Egypt.

Nahlahussein@aucegypt.edu, ORCID 0000-0003-2520-0896

Supplementary Table S1: Predicted interaction sites of wild type RcsF and IgaAperip, number of hydrogen bonds and energy of RcsF-IgaAperip conformers based on Phyre2. For convenience, amino acids residues in IgaAperip are numbered from 1 to 295, where Asp1 and Tyr295 corresponds to Asp361 and Tyr654 in the full IgaA ORF, respectively. Amino acid numbers between brackets indicate the numbering based on complete *igaA* ORF.

| Conformer | No of H, ionic and arene interactions | energy | Amino acids residues in IgaA | Amino acids in RcsF |
| --- | --- | --- | --- | --- |
| 1 | 6 | -52.84 | Asp125 (485)  Gly155 (515)  Asp159 (519)  Leu190 (550)  Thr203(563) | Lys90  Arg91  Glu68  Gln79  Ser77 |
| 2 | 1 H  1 ionic | -48.28 | Ser111 (471)  Glu202 (562) | Asp80  Arg50 |
| 3 | 1 | -47.38 | Asp222 (583) | Gln121 |
| 4 | 4 H bonds  1 ionic  1 arene interaction | -45.61 | Ala167 (527)  Ser222 (582)  Asp223 (583)  Asp230 (590)  Tyr239 (599) | Lys98  Arg91  Asn102  Asn130 |
| 5 | 1 H  1 I | -44.76 | Lys118 (478)  Gly120 (480) | Glu68  Met99 |
| 6 | 5 H  1 I | -43.13 | Glu223 (584)  Tyr599 (239)  Asp240 (600) | Glu68  Asn95  Met99  Cys118  Arg91  Lys90 |
| 7 | 3H  1 I | -42.11 | Arg89 (473)  Glu224 (584)  Leu238 (598) | Glu68  Asp72  Lys98 |
| 8 | 2 H  1 I | -40.17 | Leu132 (492)  Asp161 (521)  Lys165 (525) | Lys98  Lys90  Cys74 |
| 9 | 3 H  3 I | -39.74 | Asp161 (521)  Lys165 (525)  Asp240 (600) | Asn95  Lys98  Cys118  Lys90  Arg91 |
| 10 | 1 H  1 I | -39.63 | Ser111 (471)  Asp125 (485) | Glu110  Arg120 |
| 11 | 3 H  1 I | -39.4 | Asn168 (528)  Glu224 (584)  Asp230 (590) | Lys98  Met99  Arg91 |
| 12 | 3 H  3 I | -39.37 | Ala110 (470)  Asp136 (496) | Glu68  Lys100 |
| 13 | 2 H  3 I | -39.1 | Arg112 (473)  Lys164 (525)  Asp240 (600) | Asp80  Lys90  Arg91 |
| 14 | 1 I | -38.97 | Asp240 (600) | Lys100 |
| 15 | 2 H  1 I | -38.80 | Glu224 (584)  Asp230 (590)  Tyr239 (599) | Lys98  Lys90  Ala101 |

Supplementary Table S2: : Nucleotide sequence of *rscF*mim.

| Name | Sequence |
| --- | --- |
| *rcsF*mim | atgCGTGCTTTACCGATCTGTTTAGTAGCACTCATGCTAAGCGGCTcTgctagcCAAATCAACGCCTCTAAAATGAAAGCCAATGCTGTATTACTGCATAGCTGCGAAGTCACCAGCGGTACGCCAGGCTGCTATCGTCAGGCTGTATGTATCGGTTCTGCGCTTAACATTACGGCGAAA |

Supplementary Table S3: Predicted interaction of RcsFmim and IgaAperip based on AlphaFold2. For convenience, amino acids residues in IgaAperip are numbered from 1 to 295, where Asp1 and Tyr295 corresponds to Asp361 and Tyr654 in the full IgaA ORF. Amino acid numbers between brackets indicate the numbering based on complete *igaA* ORF. In RcsFmim, numbers between brackets indicate numbering based on wild type RcsF sequence.

| IgaAperip | RcsFmim | No. of Interactions (H-bonds) |
| --- | --- | --- |
| Arg180 (540) Ser183 (543) | Asn28 (N102) | 2 |
| Leu178 (538) | Val30 (V104) | 2 |
| Asn176 (536) | Leu32 (L106) | 2 |
| Asn156 (517) | Ser53 (127) | 1 |
| Asn153 (514) | Asn56 (130) | 1 |
